# Prospective Validation of an Ultrasound Capsule‐Based Model for Predicting Follicular Thyroid Carcinoma Without High‐Risk Features

**DOI:** 10.1002/cam4.71190

**Published:** 2025-08-29

**Authors:** Xiang‐yun Yao, Xin Li, Fang Mei, Bo Yu, Shi‐bing Song, Li‐gang Cui, Shi Tan

**Affiliations:** ^1^ Department of Ultrasound Peking University Third Hospital Beijing China; ^2^ Department of General Surgery Peking University Third Hospital Beijing China; ^3^ Department of Pathology Peking University Third Hospital, School of Basic Medical Sciences, Peking University Health Science Center Beijing China

**Keywords:** capsule, follicular thyroid carcinoma, neoplasm invasiveness, thyroidectomy, ultrasound imaging

## Abstract

**Background:**

The surgical indications for follicular thyroid neoplasms (FTNs) remain controversial due to challenges in the preoperative follicular thyroid carcinoma (FTC) diagnosis. We aimed to explore the sonographic features of the FTN capsule and establish a prediction model for diagnosing FTC without high‐risk features.

**Methods:**

This prospective cohort study enrolled consecutive adult patients with FTN. Patients presenting with extrathyroidal extension or extracapsular angioinvasion on preoperative imaging were excluded. Intraoperative ultrasound (US)‐guided incisions were conducted in 20 patients during thyroidectomy. Sonographic features of FTN capsules were identified and validated through comparison with US, macroscopic, and microscopic pathology images from the same US‐selected section. Invaded capsules were categorized based on pathological indicators of capsular invasion. The diagnostic performance of the US capsule‐based model and US risk stratification systems (American College of Radiology Thyroid Imaging Reporting and Data System [ACR‐TIRADS] and Ultrasound Follicular Thyroid Imaging Reporting and Data System [F‐TIRADS]) were compared.

**Results:**

Seventy‐four patients with unifocal lesions and 14 patients with multifocal lesions were enrolled and pathologically diagnosed with follicular thyroid adenoma (*n* = 67) and FTC (*n* = 35). As widely invasive FTC was initially excluded, there were 34 minimally invasive subtypes and 1 encapsulated angioinvasive subtype. The areas under the curves for the US capsule‐based model, ACR‐TIRADS, and F‐TIRADS were 0.839 (95% confidence interval [CI], 0.753–0.904), 0.852 (95% CI, 0.768–0.914), and 0.840 (95% CI, 0.755–0.905), respectively. No significant differences were observed in the areas under the curves, sensitivity, or accuracy of the US capsule‐based model, ACR‐TIRADS, and F‐TIRADS. The specificities of the US capsule‐based model and F‐TIRADS were higher than that of the ACR‐TIRADS (88.1% and 80.60%, respectively, vs. 44.8%, *p* < 0.05).

**Conclusions:**

Careful US scanning enables clear FTN capsule visualization, providing a straightforward, specific method for diagnosing FTC and guiding completion thyroidectomy by detecting intracapsular angioinvasion in patients with FTC.

## Graphical abstract

1. US successfully identified the capsule of follicular thyroid nodules, with discontinuity in the capsule serving as a specific indicator for follicular thyroid carcinoma (FTC).
2. Intracapsular angioinvasion could also be identified by US, showing promise for guiding completion thyroidectomy by identifying patients with encapsulated angioinvasive FTC.
3. US capsule‐based model exhibited higher specificity than ACR‐TIRADS systems, while maintaining comparable AUC values, despite lower sensitivity for certain nodule locations.

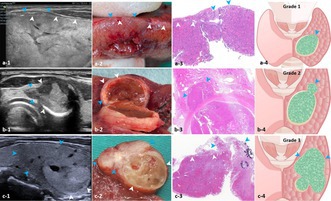

**Abbreviations**
ACR‐TIRADS: American College of Radiology Thyroid Imaging Reporting and Data System
AUC: area under the ROC curve
CEUS: contrast‐enhanced ultrasonography
CI: confidence interval
EA‐FTC: encapsulated angioinvasive follicular thyroid carcinoma
FTA: follicular thyroid adenoma
FTC: follicular thyroid carcinoma
F‐TIRADS: Ultrasound Follicular Thyroid Imaging Reporting and Data System
FTN: follicular thyroid neoplasms
MI‐FTC: minimally invasive follicular thyroid carcinoma
PTC: papillary thyroid carcinoma
ROC: receiver operating characteristic
US: ultrasound
WI‐FTC: widely invasive follicular thyroid carcinoma

## 1 Introduction

Thyroid cancer prevalence is increasing worldwide. Papillary and follicular thyroid carcinomas (FTC) constitute the majority of differentiated thyroid cancers. Compared with papillary thyroid carcinoma (PTC), FTC predominantly exhibits distant metastases through hematogenous spread, leading to a worse prognosis [1]. Unlike PTC diagnosed through nuclear features, FTC and follicular thyroid adenomas (FTA) are encapsulated follicular‐derived tumors showing cytological consistency, which results in the similarity of their ultrasound (US) imaging based on tissue acoustic impedance differences. Thus, complete removal of tumors via thyroidectomy and evaluation of capsular and vascular invasion under a microscope remain the only diagnostic methods for FTCs [2]. Individualized management of follicular thyroid neoplasms (FTN) should balance early diagnosis of FTC and avoid overtreatment for FTA.

Establishing a risk stratification system for the preoperative diagnosis of FTC is a common objective among specialists worldwide. Several risk stratification models have been proposed based on US characteristics [3, 4, 5]. However, deviations and controversies arise from the constrained nature of static US images, which is attributable to the retrospective design of the previous studies [6]. Given that the capsule of the FTN cannot be accurately located and identified using current US techniques, the preoperative diagnosis of FTC is challenging.

Benefiting from the close collaboration of the Thyroid Cancer Multidisciplinary Team at our institution, we conducted a validation test for sonographic capsule identification by correlating US images with pathological histology and photographs of specimens obtained from the corresponding US‐selected area. To date, few studies have substantiated the identification of capsules for FTN [7, 8].

The primary objective of this study was to delineate the sonographic features of capsules and capsular invasion of FTNs. The secondary objective was to compare the diagnostic performance of the capsule‐based model with those of the American College of Radiology Thyroid Imaging Reporting and Data System (ACR‐TIRADS) [3] and Ultrasound Follicular Thyroid Imaging Reporting and Data System (F‐TIRADS) [9] systems for FTC.

## 2 Materials and Methods

This prospective study was approved by the Medical Science Research Ethics Committee of our institution (Approval No: M2023722). Informed consent was obtained from all the patients.

### 2.1 Patients

**FIGURE 1:**
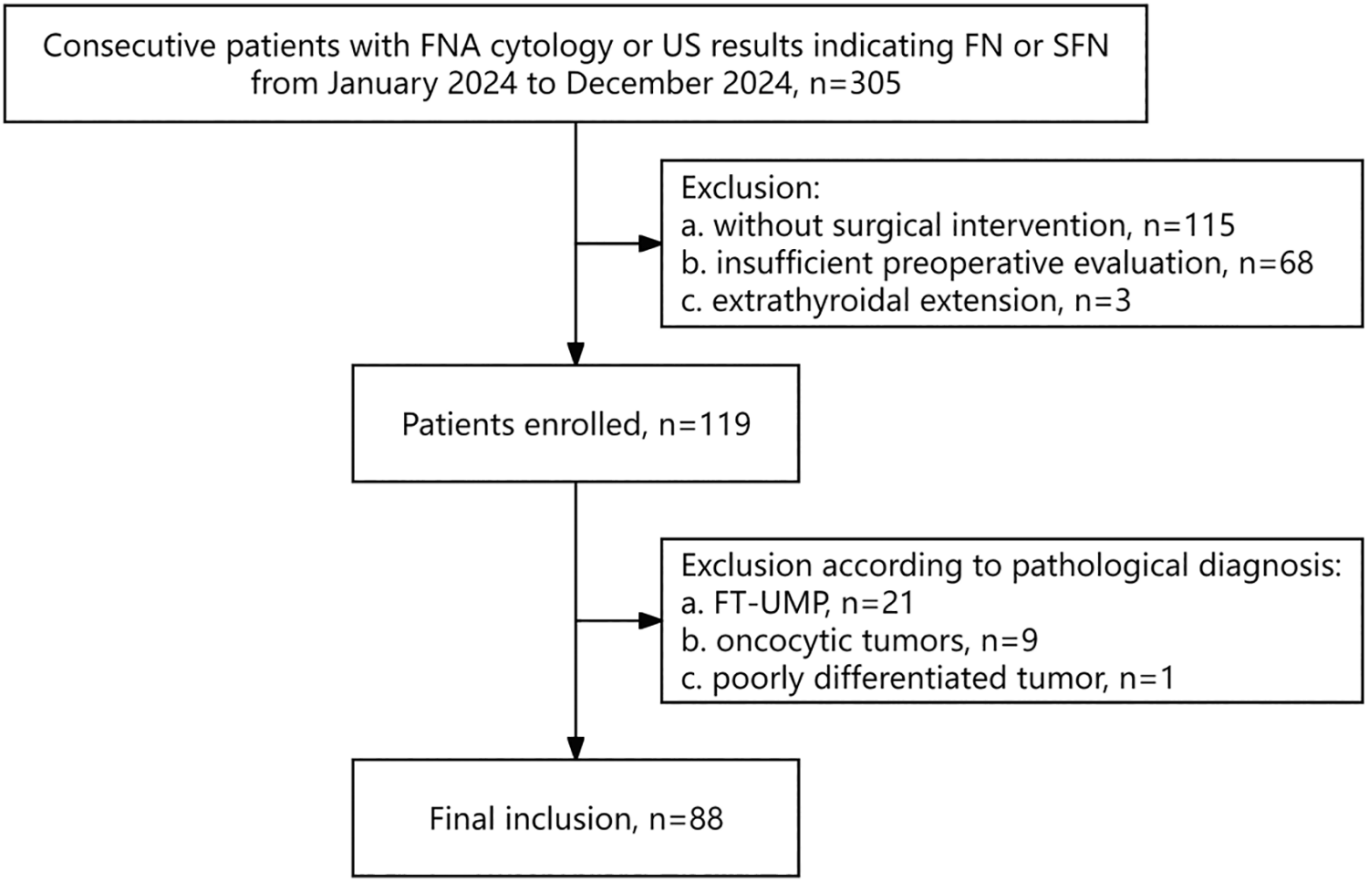
Patient flowchart. FNA, fine needle aspiration; FTN, follicular thyroid neoplasm; FT‐UMP, follicular tumor of uncertain malignant potential; SFTN, suspected follicular thyroid neoplasm.

Data from consecutive adult patients classified as having follicular neoplasm or suspected follicular neoplasm according to the Bethesda System for Reporting Thyroid Cytopathology or US examination and scheduled for thyroidectomy were collected between January 2024 and December 2024. After excluding patients diagnosed with follicular tumors of uncertain malignant potential (*n* = 21), oncocytic tumors (*n* = 9), poorly differentiated tumors (*n* = 1), and PTC (*n* = 1), 88 patients were ultimately included. Given that some patients had multiple lesions, 35 cases of pathologically confirmed FTC and 67 cases of pathologically confirmed FTA were ultimately included in the study (Figure 1).

### 2.2 Collection of US Imaging Data

Three radiologists with over 5 years of experience performed the thyroid US examination. The US devices used in this study included RS80 (Samsung), Resona7 (Mindray), and Ultimus9E (Vinno), each equipped with 5‐ to 12‐MHz or 4‐ to 18‐MHz linear‐array transducers. Static and dynamic US images of transverse and longitudinal sections of each nodule were obtained. Following the conventional thyroid US examination, the initial contour was delineated through a full scan of the nodules. Subsequently, the focus shifted to assessing the integrity or disruption of the tumor contour on US. The margin areas of the FTNs were zoomed in and emphasized during scanning to acquire high‐resolution images of the marginal areas of the collected tumors.

### 2.3 Validation Test of Capsule Visualization by US

Validation tests were performed on 20 patients who underwent intraoperative US examination using an M9 US System (Mindray, Shenzhen, China) equipped with a 5‐ to 16‐MHz hockey linear‐array transducer. The transducer was coated with acoustic gel, covered with a sterile sleeve, and placed on the exposed thyroid lobes. The specimens were excised during thyroidectomy, with the site and angle of incision guided by intraoperative US to ensure precise alignment of the US section with the macroscopic and microscopic pathology. Sections with lobulated margins, or local protrusions on US were selected. The maximum diameter section was chosen for nodules with smooth margins. The exported images of US and correlating photographs of specimens and pathological histology data obtained from the same US‐selected section were imported into AutoCAD version 2015 (Autodesk Corporation, California, USA).

### 2.4 Features of Capsular and Intracapsular Vascular Invasion Assessed by US

**FIGURE 2:**
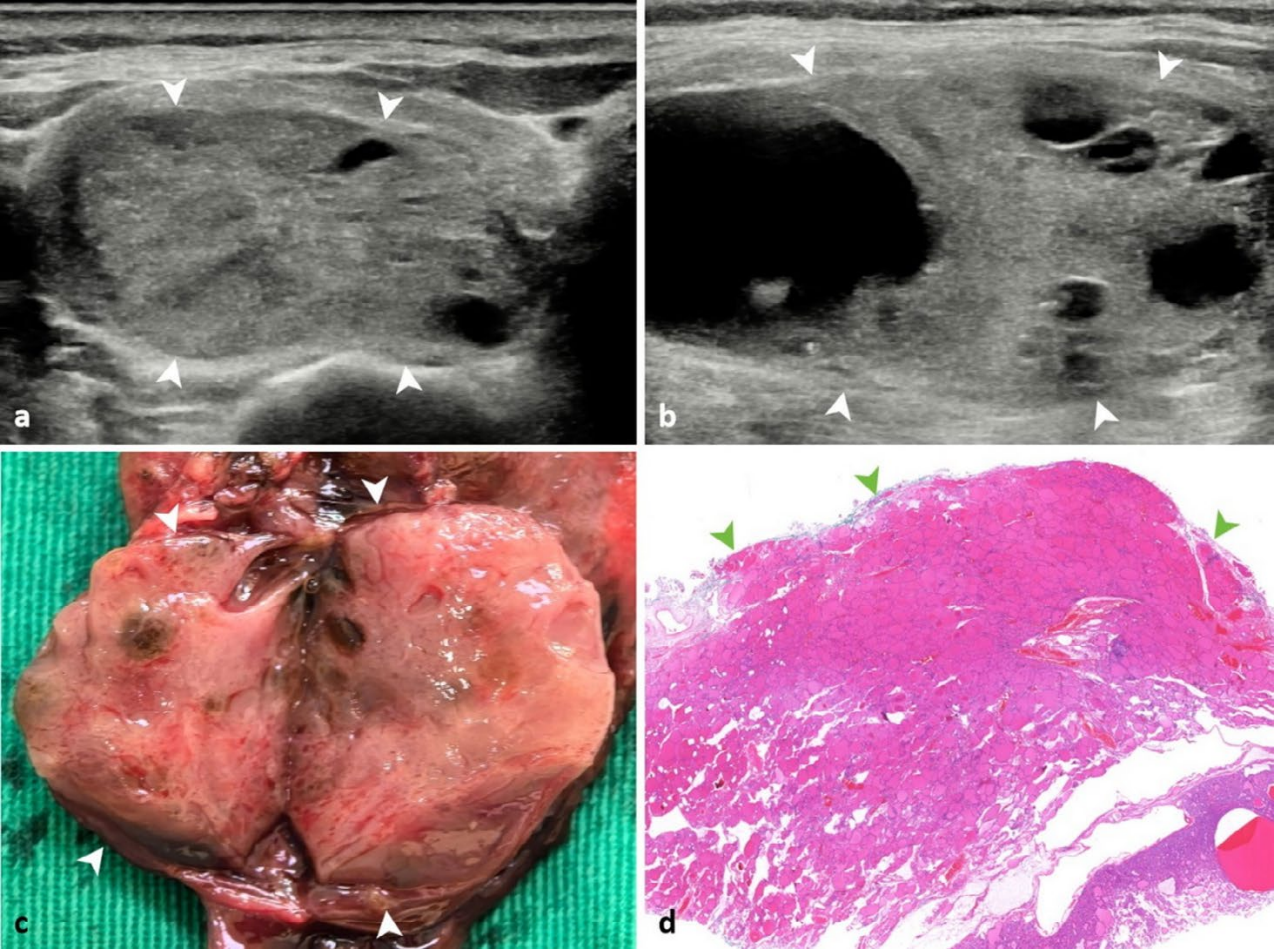
Sonographic and pathological features of the intact FTN capsule (grade 0). (a, b) Ultrasound images demonstrate a thin hyperechoic rim surrounding the nodule (arrows). (c) Gross specimen and (d) histopathological section confirm that the hyperechoic line on ultrasound corresponds to the fibrous capsule (arrows) of the FTN (hematoxylin and eosin, 2× magnification). FTN, follicular thyroid neoplasm. FTN, follicular thyroid neoplasm.

**FIGURE 3:**
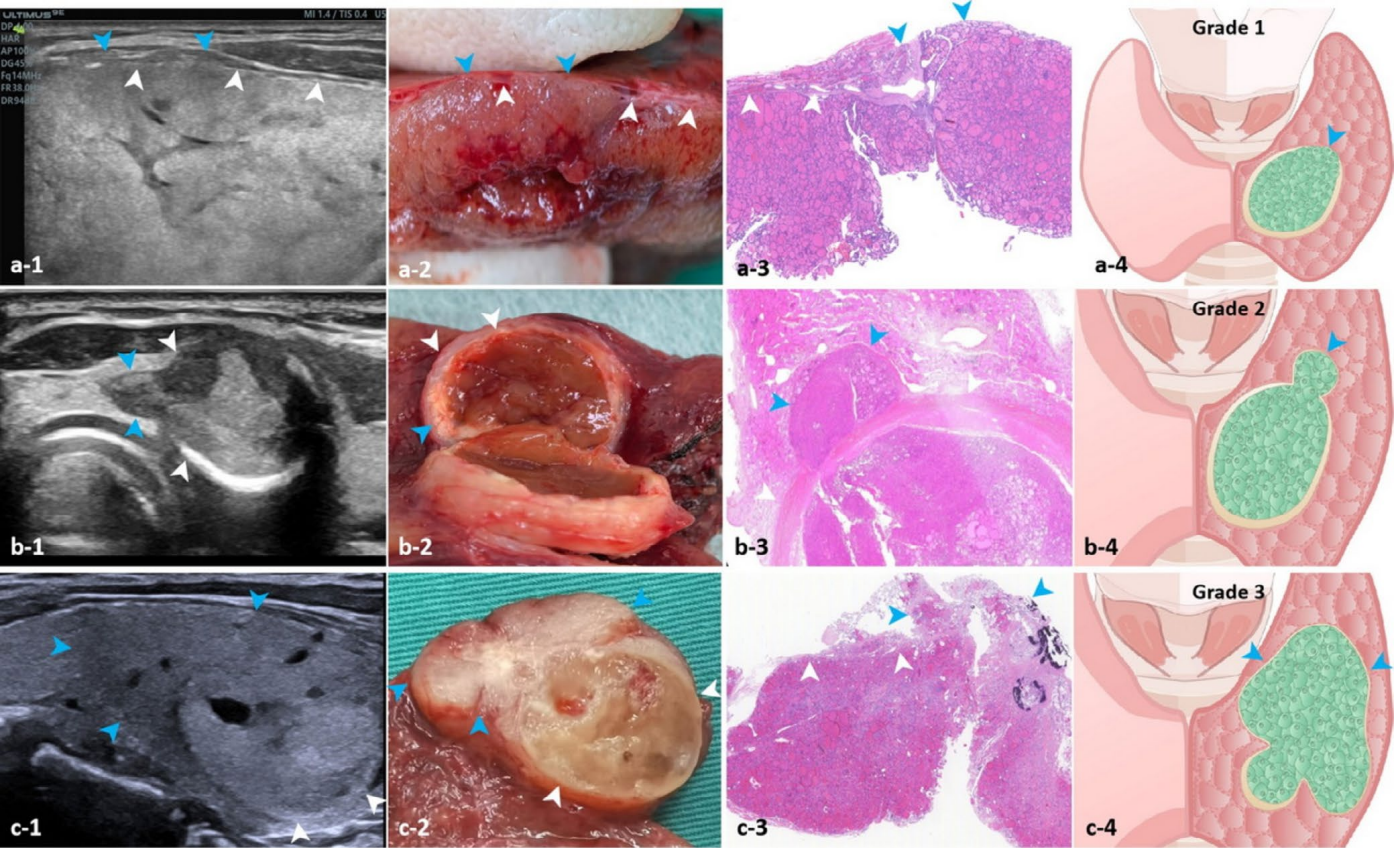
Sonographic and pathological features of the capsule‐based model. (a) The invaded capsule appears as a discontinuity (blue arrows) of the hyperechoic line (white arrows); (b) The invaded capsule appears as a local tumor protrusion resembling a mushroom (blue arrows). The nodule demonstrates eggshell calcification, and the capsule corresponds to the eggshell (white arrows) by comparing the thickness of the capsule between sonographic and histological images; (c) Only a minority part of the capsule can be identified (white arrows), the tumor appears as extracapsular invasion with a fibrous capsule (blue arrows) (a‐3, b‐3, c‐3: Hematoxylin and eosin, 1× magnification).

After adjusting the angle of the images, the capsules of FTN were identified on the pathological images. Subsequently, the distance between the FTN capsule and specimen margin was calculated. Finally, by delineating a line equivalent to the aforementioned distance from the thyroid gland margin on the US image, it was observed that the intact capsule corresponded to the hyperechoic line of the margin or the inner layer of the halo (Grade 0, Figure 2). Conversely, the invaded capsules were seen as disruptions in the hyperechoic line (Grade 1, Figure 3a), local tumor protrusions resembling mushroom‐like shapes or satellite nodules (Grade 2, Figure 3b), or extracapsular invasion marked by a fibrous capsule (Grade 3, Figure 3c).

**FIGURE 4:**
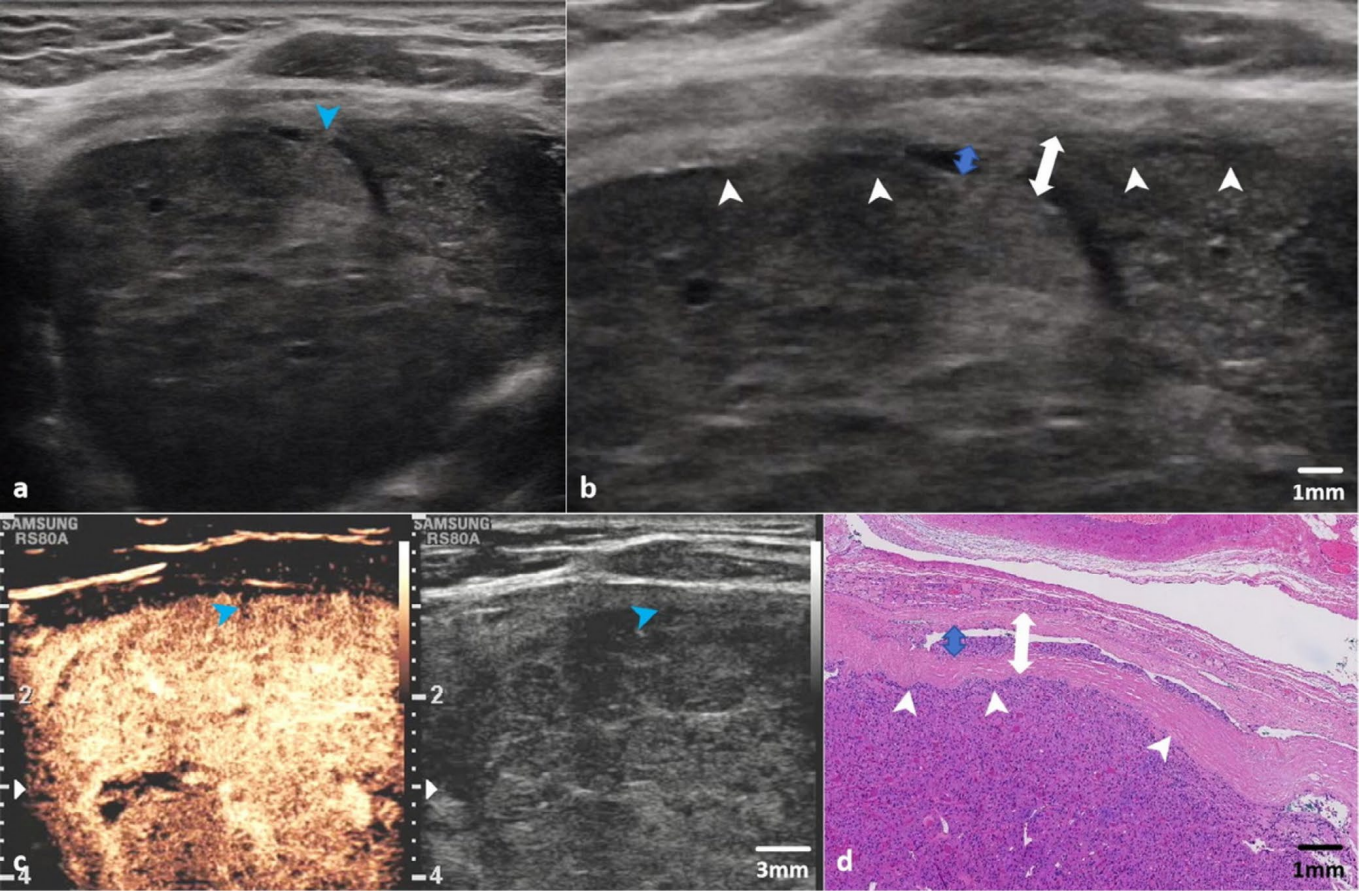
Intracapsular vascular invasion identified by US and CEUS. Images from a 59‐year‐old female patient with pathologically confirmed EA‐FTC. (a, b) Conventional US shows a tumor embolus (blue arrows) in the margin of the tumor (white arrows). (c) After the patient's consent was obtained, perfluorobutane microbubbles (Sonazoid, GE HealthCare; 0.01 mL/kg) were administered intravenously. CEUS demonstrates no enhancement within the vessel (blue arrow). (d) Histological image confirms intracapsular vascular invasion (blue arrow) within the capsule (white double‐headed arrow) (hematoxylin and eosin, 4× magnification). CEUS, contrast‐enhanced ultrasonography; EA‐FTC, encapsulated angioinvasive follicular thyroid carcinoma; US, ultrasound.

The status of intracapsular vessels was evaluated. Dilated angioid structures within the capsule were deemed indicative of potential intracapsular angioinvasion. Contrast‐enhanced ultrasonography (CEUS) was conducted to confirm the presence of a tumor thrombus in intracapsular vessels. The characteristics of the intracapsular angioinvasion are illustrated in Figure 4.

### 2.5 Evaluation of US Images

Two radiologists with more than 5 years of experience in thyroid US independently reviewed the US images and videos. Disagreements between radiologists were resolved by a senior radiologist with > 15 years of experience in thyroid US. All reviewers were blinded to clinical information and pathological results.

### 2.6 Categorization of Follicular Nodules

The nodules were scored on the basis of US characteristics according to the ACR‐TIRADS (3) and F‐TIRADS (9) systems. Nodules scoring 4 points and ≥ 7 points were categorized as having an intermediate and high risk of FTC according to the ACR‐TIRADS and F‐TIRADS systems, respectively. The others were classified as having a low risk for FTC. Based on the capsule‐based model, nodules with capsular invasion graded 1–3 or intracapsular angioinvasion were considered to have an intermediate and high risk of FTC, whereas those with intact capsules (grade 0) were considered to have a low risk of FTC.

### 2.7 Statistical Analysis

Using the Poisson distribution method, the minimum sample size required to detect capsular or vascular invasion was 58 (rate [*π*], 0.8; power, 0.8; *λ*, 3).

Statistical analyses were conducted using IBM SPSS software (version 23.0, IBM, Armonk, NY, USA). Continuous variables were presented as mean (standard deviation) or median (range) according to the distribution. Categorical variables were presented as numbers and percentages. Differences between groups were analyzed using an independent two‐sample *t*‐test or Mann–Whitney *U* test for continuous variables, and a chi‐square test for categorical variables. The diagnostic performance of the systems was evaluated using a receiver operating characteristic (ROC) curve analysis. The sensitivity, specificity, and accuracy were calculated, and the area under the ROC curve (AUC) was reported with 95% confidence intervals (CIs). Statistical significance was defined as a two‐sided *p*‐value < 0.05. Differences between the AUCs of the systems were compared using the DeLong test in MedCalc software (version 20.0, MedCalc Software Ltd., Ostend, Belgium).

## 3 Results

### 3.1 Clinical Characteristics

The study population comprised 11 male (12.5%) and 77 female (87.5%) patients. The mean age of the patients was 52.6 ± 14.4 years (range, 22–79 years). Subsequent to thyroidectomy, histopathological examination confirmed 67 (65.7%) FTAs and 35 (34.3%) FTCs. Among the FTC subtypes identified, 34 (97.1%) were classified as minimally invasive (MI‐FTC) and 1 (2.9%) as encapsulated angioinvasive (EA‐FTC). The mean maximum diameter of the FTNs was 3.7 ± 1.8 cm (range, 0.6–11.6 cm).

### 3.2 Clinical and US Features Associated With FTC

There were no significant differences between FTA and FTC with respect to age, sex, maximum diameter, or composition.

Compared with FTA, FTC showed a tendency to be hypoechoic (51.4% vs. 26.9%, *p* = 0.01), lobulated or irregular margins (85.7% vs. 35.8%, *p* < 0.01), and an uneven thickness of the peripheral halo (45.7% vs. 10.4%, *p* < 0.01), and exhibited macro or rim calcifications (60.0% vs. 19.4%, *p* < 0.01), trabecular formation (34.3% vs. 6.0%, *p* < 0.01), and “nodule in nodule” signs (14.3% vs. 3.0%, *p* = 0.045) (Table 1).

**TABLE 1:** Comparison of the ultrasound features of follicular thyroid carcinoma and follicular thyroid adenoma.

Parameter	FTA (*n* = 67)	FTC (*n* = 35)	*p*
Age (years)	53.5 ± 13.7	52.2 ± 14.7	0.650
Sex (*n*)			
Male	10	5	0.931
Female	57	30	
Maximum diameter (cm)	3.5 ± 1.5	4.1 ± 2.2	0.121
Composition (*n*)			
Solid	39	25	0.257
Predominantly solid	25	10	
Predominantly cystic	3	0	
Echogenicity (*n*)			
Hypoechoic	18	18	0.014
Isoechoic	49	17	
Margin (*n*)			
Smooth	43	5	< 0.01
Lobulated or irregular	24	30	
Calcifications (*n*)			
Absent	49	8	< 0.01
Microcalcification	5	6	
Macrocalcification	12	14	
Rim calcification	1	7	
Halo (*n*)			
Absent	44	14	< 0.01
Uniform halo	16	5	
Uneven halo	7	16	
Trabecular formation (*n*)			
Absent	63	23	< 0.01
Present	4	12	
“Nodule in nodule” sign (*n*)			
Absent	65	30	0.045
Present	2	5	

Abbreviations: FTA, follicular thyroid adenoma; FTC, follicular thyroid carcinoma.

### 3.3 Diagnostic Performance of Risk Stratification Systems

Based on the capsular evaluation results obtained by US, 1 nodule was classified as intracapsular angioinvasion, 12 as grade 1, 8 as grade 2, and 12 as grade 3. Utilizing grades 1–3 and intracapsular angioinvasion as the threshold values, there were 10 false negatives and 8 false positives. The sensitivity, specificity, and accuracy of the capsule‐based model were 71.4% (25/35), 88.1% (59/67), and 82.4% (84/102), respectively.

The ACR‐TIRADS system categorized 70 nodules as high‐ and intermediate‐risk. There were 2 false negatives and 37 false positives. The sensitivity, specificity, and accuracy of the ACR‐TIRADS were 94.3% (33/35), 44.8% (30/67), and 61.8% (63/102), respectively.

The F‐TIRADS system categorized 41 nodules as high or intermediate risk. There were 7 false negatives and 13 false positives. The sensitivity, specificity, and accuracy of the F‐TIRADS were 80.0% (28/35), 80.6% (54/67), and 80.4% (82/102), respectively.

No significant differences were observed between the sensitivities and accuracies of these systems (*p* > 0.05). The specificities of the capsule‐based model and F‐TIRADS were significantly higher than that of the ACR‐TIRADS (*p* < 0.01 and *p* = 0.01, respectively).

**FIGURE 5:**
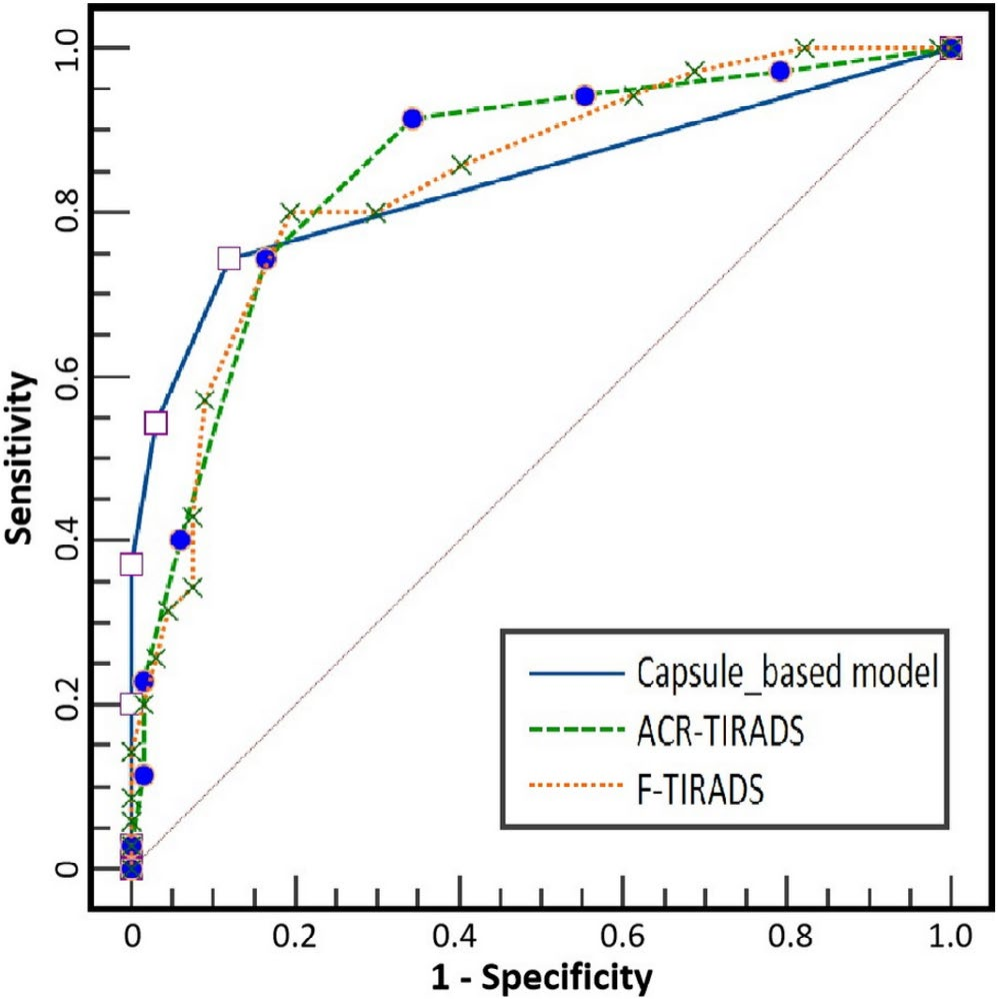
Receiver operating characteristic curves of the ACR‐TIRADS, F‐TIRADS, and capsule‐based model. ACR‐TIRADS, American College of Radiology Thyroid Imaging Reporting and Data System; F‐TIRADS, Ultrasound Follicular Thyroid Imaging Reporting and Data System.

The AUCs for the capsule‐based model, ACR‐TIRADS, and F‐TIRADS were 0.839 (95% CI: 0.753–0.904), 0.852 (95% CI: 0.768–0.914), and 0.840 (95% CI: 0.755–0.905), respectively (Figure 5).

There were no significant differences among the AUCs of the three systems (*p* > 0.05) (Table 2).

**TABLE 2:** Comparison of the diagnostic performance of the capsule‐based model and other risk stratification systems.

Parameter	Cutoff criterion	Sensitivity	Specificity	Accuracy	AUC	95% confidence interval	*p*_AUC_
Capsule‐based model	Grade ≥ 1	71.4%	88.1%	82.4%	0.839	0.753–0.904	Ref
ACR‐TIRADS	≥ 4 points	94.3%	44.8%	61.8%	0.852	0.768–0.914	0.752
F‐TIRADS	≥ 7 points	80.0%	80.6%	80.4%	0.840	0.755–0.905	0.966

Abbreviations: ACR‐TIRADS, American College of Radiology Thyroid Imaging Reporting and Data System; AUC, area under the receiver operating characteristic curve; F‐TIRADS, Ultrasound Follicular Thyroid Imaging Reporting and Data System; *p*_AUC_, *p*‐value for AUC through DeLong test; US, ultrasound.

## 4 Discussion

This study visualized FTN histopathology utilizing US and confirmed the consistency between sonographic features and histopathology. Owing to variations in transducer frequency and acoustic beam focus when evaluating capsules of different sizes of FTN nodules, results from static images obtained by conventional thyroid US examinations were limited in scope for the study's objective. To the best of our knowledge, few studies have differentiated between benign and malignant FTNs using prospectively collected images. The prospectively acquired materials herein yielded comprehensive imaging data for each FTN included and facilitated the detailed FTN capsule visualization.

**FIGURE 6:**
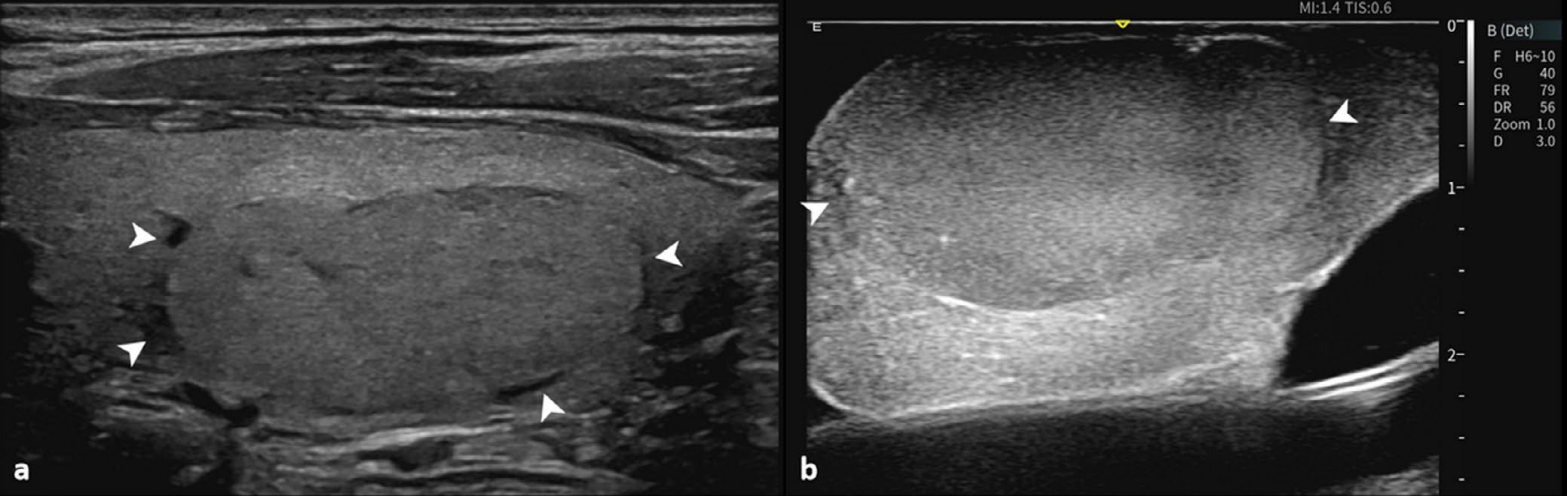
Comparison of thickness of the nodule's halo before and after nodule resection. (a) Conventional ultrasound image reveals an uneven halo of the nodule (arrows). (b) Intraoperative ultrasound demonstrates a reduction in thickness of the nodule's halo (arrows), following the resection of the thyroid lobe. The attenuation of the halo's thickness is attributed to the blockage of the thyroid arteries.

Several indicators associated with FTN capsules have been proposed, including margin, halo, and extrathyroidal extension [10, 11, 12]. Although lobulated or irregular margins are associated with FTC, with capsular invasion, the margin of FTNs is also determined by the relative pressure between the nodule and surrounding thyroid parenchyma. Because of the complex anatomy of its neck and tissue structure, the thyroid gland experiences variable pressure throughout. This corresponds to a certain proportion of the FTAs with lobulated margins. The diagnostic value of halos has decreased recently. The prevailing view of the halo composition is that it consists of compressed vessels around thyroid nodules [13]. Our experience at a single center confirmed this opinion by revealing a noticeable attenuation in the thickness of the halo when the thyroid arteries were blocked during thyroidectomy (Figure 6). However, some studies have associated the variable thickness of the peripheral halo with FTCs. We hypothesized that the invasion of FTC capsules and vessels leads to the thickening of capsules [14], decreasing the echogenicity of the surrounding thyroid parenchyma.

Although several sonographic features were associated with FTC, most parameters were evaluated subjectively. To standardize the evaluation process and sonographic features, with reference to the gold standard for pathological diagnosis of FTC, we endeavored to identify the FTN capsule on US in initial clinical practice. Beginning with an FTA exhibiting a predominantly cystic composition, an intact, thin, hyperechoic line was consistently observed surrounding the nodules. However, the capability of US to identify the FTN capsule has remained a persistent question. The present study represents the first attempt to verify US identification of FTN capsules utilizing intraoperative procedures. During thyroidectomy, the lesion was placed in a container filled with normal saline after the removal of the thyroid lobes. Benefiting from the proximity between the intraoperative ultrasonic probe and FTNs, the frequency of the transducer can be set higher, and the angle of the transducer can be adjusted freely for specimens in vitro. Under intraoperative US guidance, the precise location and orientation of the incision for specimen extraction were meticulously coordinated with imaging findings on US. Thus, ultrasonic features of FTN capsules can be confirmed by matching US images with the capsule identified under macroscopic and microscopic pathology in the same US‐selected section. Based on the identification and verification of the hyperechoic linear margin of the inner layer of the halo as the FTN capsule, the presence of a discontinuity in the capsule was initially regarded as a suspicious US characteristic of FTC. Furthermore, referring to the microscopic pathological features of capsular invasion, additional ultrasonographic features were identified as follows: local tumor protrusion resembling a mushroom‐like shape or satellite nodule, and extracapsular invasion with fibrous capsule.

With conventional US features, three additional signs, namely rim calcification [9], trabecular formation [10], and “nodule in nodule” sign [15], indicate FTC. The incidences of the three signs were 20.0% (7/35), 34.3% (12/35), and 14.3% (5/35), respectively. Although frequencies of these signs were lower than those of other features, corresponding to previous studies, the high specificity deserves special attention during the US examination of FTNs. In contrast to the disruption of capsules caused by local capsular invasion and tumor protrusion, the three signs may arise from interactions between the thyroid parenchyma and invasive tumor tissues [16, 17]. Calcification and fibrosis of thyroid tissue can result in rim and trabecular calcification, respectively. The protruded tumor could form new nodules, forming a “nodule in nodule” sign or a “daughter nodule” next to the “mother nodule.”

No significant differences were observed in AUCs among the ACR‐TIRADS, F‐TIRADS, and capsule‐based model. Without regard to diameter, echogenicity, composition, and calcifications, with discontinuity of the capsule as the only predictor for FTC, the specificity of the capsule‐based model was higher than that of ACR‐TIRADS. The relatively lower sensitivity of the capsule‐based model may be attributed to two main factors: (1) the extent of capsular invasion is often minimal and only microscopically detectable in some cases, and (2) ultrasound imaging has inherent limitations in evaluating substernal nodules and lateral capsular features. Nonetheless, the US capsule‐based model demonstrated diagnostic performance comparable to those of both ACR‐TIRADS and F‐TIRADS systems. With the capacity to describe capsular invasion, intracapsular angioinvasion could be demonstrated by US and confirmed by CEUS. As with the widely invasive FTC (WI‐FTC), the EA‐FTC subtype is similarly associated with poor prognosis. Consequently, completion thyroidectomy is recommended for these two subtypes of FTC according to the current consensus [18, 19]. However, unlike the well‐established sonographic features of extrathyroidal extension of WI‐FTC, features of EA‐FTCs were previously considered undetectable on conventional ultrasonography. Therefore, the capsule‐based model presents a promising approach for reducing the necessity of secondary surgery caused by discrepancies between frozen pathology and paraffin pathology in patients with EA‐FTC. This further highlights the significance of surgeon‐sonographer collaboration in ultrasound examinations for guiding surgical decision‐making [20].

This study has a few limitations. First, the sample size is small. Second, only patients planning to undergo thyroid surgery were included, potentially resulting in a selection bias. Third, only one patient with encapsulated angioinvasive FTC was included because of its relatively low incidence compared with that of the minimally invasive subtype. Finally, capsule visualization via sonography was inevitably influenced by the tumor size and substernal location in some cases. Future studies with larger patient cohorts are required to allow for stratification analyses.

In conclusion, this study proposed and validated capsule visualization utilizing US. The capsule evaluation via US provides a singular, direct indicator for FTC diagnosis with comparable diagnostic efficacy to the ACR‐TIRADS and F‐TIRADS systems; it also enables the visualization of intracapsular angioinvasion. Application of CEUS in conjunction with conventional US shows promise for guiding completion thyroidectomy by identifying patients with EA‐FTC.

## Author Contributions

**Xiang‐yun Yao:** conceptualization (equal), data curation (equal), investigation (lead), writing – original draft (equal). **Xin Li:** data curation (equal), investigation (equal), resources (lead), writing – original draft (equal). **Fang Mei:** data curation (equal), methodology (lead), resources (equal), validation (equal), visualization (lead). **Bo Yu:** formal analysis (equal), investigation (equal), software (lead), visualization (equal). **Shi‐bing Song:** resources (equal), validation (equal), writing – review and editing (equal). **Li‐gang Cui:** methodology (equal), project administration (equal), resources (equal), supervision (equal), writing – review and editing (equal). **Shi Tan:** funding acquisition (lead), project administration (lead), supervision (lead), writing – review and editing (equal).

## Ethics Statement

This prospective cohort study was approved by the Medical Science Research Ethics Committee of Peking University Third Hospital (Approval No: M2023722).

## Consent

Informed consent was obtained from all individual participants included in the study.

## Conflicts of Interest

The authors declare no conflicts of interest.

## Data Availability

The datasets used and/or analyzed during the current study are available from the corresponding author on reasonable request.
